# Mendelian Randomization Rules Out Causation Between Inflammatory Bowel Disease and Non-Alcoholic Fatty Liver Disease

**DOI:** 10.3389/fphar.2022.891410

**Published:** 2022-05-19

**Authors:** Lanlan Chen, Zhongqi Fan, Xiaodong Sun, Wei Qiu, Yuguo Chen, Jianpeng Zhou, Guoyue Lv

**Affiliations:** Department of Hepatobiliary and Pancreatic Surgery, The First Hospital of Jilin University, Changchun, China

**Keywords:** null association, causal inference, genetic epidemiology, mendelian randomization, NAFLD (non alcoholic fatty liver disease), IBD—inflammatory bowel disease

## Abstract

**Background:** Inflammatory bowel disease (IBD) and non-alcoholic fatty liver disease (NAFLD) usually co-exist clinically. However, whether such association is causal is still unknown.

**Methods:** Genetic variants were extracted as instrumental variables from the largest genome-wide association study (GWAS) of IBD, Crohn’s disease (CD) and ulcerative colitis (UC) with 25,042 cases and 34,915 controls (GWAS *p*-value < 5 × 10^−8^). Information of genetic variants in NAFLD was extracted from a GWAS with 1,483 cases and 17,781controls. Also, liver fat content (LFC) was included as the outcome. Then, a bi-direction Mendelian randomization (MR) was carried out to appraise the causal relationship between NAFLD on IBD. Besides, a multivariable MR (MVMR) design was carried to adjust for body mass index (BMI) and type 2 diabetes (T2D) as well.

**Results:** Generally, IBD might not affect the risk of NAFLD (OR = 0.994 [0.970, 1.019]), together with its subtypes including UC and CD. However, genetically-elevated risk of IBD might cause liver fat accumulation (beta = 0.019, *p*-value = 0.016) while turning insignificant at Bonferroni correction. Besides, no causal effect of NAFLD on IBD was observed (OR = 0.968 [0.928, 1.009]), together with UC and CD. Also, genetically-elevated LFC could not impact IBD, UC and CD either. The MR CAUSE analysis supported these null associations and MVMR analysis also supported such null associations even after adjusting for BMI and T2D.

**Conclusion:** This MR study ruled out the causal relationship between IBD and NAFLD, suggesting therapeutics targeting NAFLD might not work for IBD and vice versa.

## Introduction

Non-alcoholic fatty liver disease (NAFLD), a disease characterized with liver steatosis and determined by liver fat content (LFC), is amongst the most important causes of liver diseases even in lean patients and its global prevalence is estimated to reach over 24% (Younossi et al., 2018). Inflammatory bowel disease (IBD), including ulcerative colitis (UC) and Crohn’s disease (CD), is a chronic intestinal inflammation which can reduce patient’s life expectancy from age-related comorbidities like cardiovascular diseases, and its global prevalence will be as high as 1% by 2030 in many regions (Kaplan and Windsor, 2021). Considerable epidemiological evidence has linked these two diseases together where IBD was associated with increased risk of NAFLD and they usually coexist (McHenry et al., 2019; Zou et al., 2019; Lin et al., 2021). However, an observational study suggested there were no significant differences in terms of IBD characteristics between IBD patients with and without NAFLD, indicating the interplay between NAFLD and IBD is no easy (Magrì et al., 2019). Besides, it should be noted that all the available evidence is based on observational studies, which might be biased by unavoidable potential confounders and reverse causation (Piovani et al., 2021). There is a paucity of evidence illustrating whether the observed association is causal.

Mendelian randomization (MR) is an emerging epidemiological method of causal inference and has made great contribution to detection of causal risk factors for diseases. For instance, Voight et al. ruled out the possibility that high-density lipoprotein cholesterol (HDL-C) could lower risk of myocardial infarction using MR design, challenging the traditional concept (Voight et al., 2012). MR design utilizes genetic variants as instrument variables (IVs), usually single nucleotide polymorphisms (SNPs) and can largely evade bias caused by potential confounders as SNPs are allocated randomly at conception and free from influence of confounders (Davey Smith and Hemani, 2014). Thanks to the rapid development of genome-wide association study (GWAS) and accumulation of publicly available GWAS summary statistics, MR design based on two-sample setting is becoming more flexible and accessible. Several MR studies have identified causal risk factors of IBD, such as body fat percentage (Carreras-Torres et al., 2020) and ankylosing spondylitis (Cui et al., 2020). Also, a MR study clarified the causal relationship between NAFLD, type 2 diabetes (T2D) and obesity (Liu et al., 2020).

However, there is no MR study exploring the causal relationship between NAFLD and IBD. In this study, we aim to explore the causal relationship between NAFLD and IBD, hoping to disentangle their complex interplay and provide useful advice in clinical practice.

## Materials and Methods

### GWAS Summary Statistics of NAFLD and IBD

The GWAS summary statistics of NAFLD were obtained from a recent published GWAS, with 1,483 European NAFLD cases and 17,781 genetically matched controls, and this study included the first five principal components as covariates (Anstee et al., 2020). Considering NAFLD is closely associated with LFC, we also selected a recent LFC GWAS which included 32,858 European participants from United Kingdom Biobank and adjusted for age at imaging visit, age squared, sex, imaging center, scan date, scan time, genotyping batch, and genetic relatedness (Liu et al., 2021). The IBD data were downloaded from an IBD meta-GWAS which included a total of 59,957 subjects, with 12,194 Crohn’s disease and 12,366 ulcerative colitis, and this study adjusted for the first ten principal components for each cohort (de Lange et al., 2017). As for the IBD GWAS, there were 25,042 European and unknown ancestry cases, together with 34,915 European and unknown ancestry controls (de Lange et al., 2017). Genomic control has been applied to all these studies. Each GWAS has been approved by corresponding Ethics Committees.

### Mendelian Randomization Design

**FIGURE 1:**
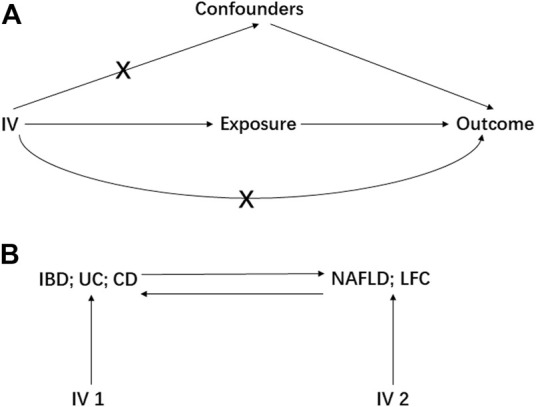
The basic principles of Mendelian randomization (MR) study. A represents the three principal assumptions; B represents the bi-direction MR design. IV is instrumental variable; IBD is inflammatory bowel disease; UC is ulcerative colitis; CD is Crohn’s disease; NAFLD is non-alcoholic fatty liver disease; LFC is liver fat content.

MR study should be carried out under three principal assumptions: (Younossi et al., 2018): the genetic variants should be closely associated with the exposure; (Kaplan and Windsor, 2021) the genetic variants should not be associated with any potential confounders that might mediate the way from exposure to outcome; (McHenry et al., 2019) the genetic variants should not be associated with outcome if conditioned on exposure (Emdin et al., 2017) (Figure 1). Besides, additional assumptions should be satisfied as well, such as linearity and no interaction between mediator and outcome.

Genetic variants were selected as IVs if reaching the genome-wide significance (GWAS *p*-value < 5 × 10^−8^) and were further clumped based on linkage disequilibrium (LD, *r* ^2^ = 0.01) and genomic region (clump window 1,000 kilobases). Also, SNP with lower minor allele frequency (MAF <0.01) would be removed in the following analysis. In the two-sample setting, we harmonized summary statistic data to ensure each IV was aligned with the same effect allele.

Preliminarily, the IBD, UC and CD were treated as the exposures to estimate their causal effect on NAFLD and LFC, resulting in six pairs of causal relationships including IBD-NAFLD, IBD-LFC, UC-NAFLD, UC-LFC, CD-NAFLD and CD-LFC. Then, a bi-directional MR analysis was performed where the NFALD and LFC were set to be the exposures, generating another six pairs of causal relationship namely NAFLD-IBD, NAFLD-UC, NAFLD-CD, LFC-IBD, LFC-UC and LFC-CD (Figure 1). Furthermore, a multivariable MR (MVMR) design was elaborated with adjusting for body mass index (BMI) and type 2 diabetes (T2D), two potential confounders according to a recent meta-analysis (Lin et al., 2021). Therein, the GWAS summary statistics of BMI was from the Genetic Investigation of ANthropometric Traits (GIANT) consortium (Locke et al., 2015) and these of T2D was from the DIAbetes Genetics Replication And Meta-analysis (DIAGRAM) consortium (Mahajan et al., 2014).

### Statistical Analysis and Data Visualization

Initially, Wald ratio estimation was utilized to obtain the effect size of exposure on outcome for each IV and then each IV’s causal effect size was combined using an inverse-variance weighted (IVW) method. F statistics were calculated for each IV to ensure a sufficient power. The Cochrane’s Q value was calculated to appraise heterogeneity and the multiplicative random effect model would be adopted if there exists heterogeneity. The MR Steiger test has been performed to judge whether the IVs affect exposure more than outcome and we would eliminate the IV if it explained outcome more than exposure (Hemani et al., 2017).

Considering the horizontal pleiotropy can largely mislead the MR estimation, various methods have been utilized to minimize the bias caused by it, including both correlated and uncorrelated horizontal pleiotropy. For correlated horizontal pleiotropy, MR-Egger regression (Bowden et al., 2015) and MR-PRESSO (Verbanck et al., 2018) were utilized. The MR-Egger regression uses the intercept obtained from regression analysis to judge the correlated horizontal pleiotropy and we assume there is no correlated horizontal pleiotropy if the intercept equals to zero (Bowden et al., 2015). The MR-PRESSO uses distortion test to detect outliers that might manifest horizontal pleiotropy and further corrects the IVW estimation with removal of outliers (Verbanck et al., 2018). For uncorrelated horizontal pleiotropy, another two methods were adopted, including weighted median (Bowden et al., 2016) and CAUSE (Morrison et al., 2020). Therein, CAUSE can allow for both correlated and uncorrelated horizontal pleiotropy, and it was functioned based on full summary statistics (Morrison et al., 2020). In CAUSE analysis, the threshold of SNP-exposure *p*-value was 1 × 10^−3^, ensuring enough IVs to estimate nuisance parameters.

All statistical analyses were performed using R packages, including “TwoSampleMR” (Hemani et al., 2018), “MRPRESSO” (Verbanck et al., 2018)and “cause” (Morrison et al., 2020) in R software 3.6.0 (https://www.r-project.org/). The data visualization was conducted using R packages, including “TwoSampleMR” (Hemani et al., 2018) and “forestplot”.

### Sensitivity Analysis

The leave-on-out sensitivity analysis was performed to find the IV that might drive the main results, guaranteeing the MR results were robust.

## Results

Generally, our MR study indicated there might be no causal relationship between NAFLD and IBD although the genetic liability to IBD might elevate the LFC slightly. The number of IVs for each phenotype varied from 4 to 145 and each F statistic was greater than the empirical threshold 10, indicating less bias caused by weak instruments (Table 1).

**TABLE 1:** A brief description of each GWAS summary statistics.

Phenotype	Ancestry	Sample Size	Unit	NSNP	R2 (%)	F	PMID
Inflammatory bowel disease	Mixed	25,042 cases and 34,915 controls	1-unit of logOR	145	15.37	74.91	28,067,908
Ulcerative disease	Mixed	12,366 cases and 33,609 controls	1-unit of logOR	75	8.53	57.07	28,067,908
Crohn’s disease	Mixed	12,194 cases and 28,072 controls	1-unit of logOR	113	12.97	52.95	28,067,908
Nonalcoholic fatty liver disease	European	1,483 cases and 17,781 controls	1-unit of logOR	4	1.59	77.79	32,298,765
Liver fat content	European	32,858 participants	SD	13	4.32	114.07	34,128,465

NSNP, the number of single nucleotide polymorphism; R2, variance of phenotype explained by SNPs; logOR, logarithm of odds ratio; SD, standard deviation; F, F statistics; PMID, ID of publication in the PubMed.

**FIGURE 2:**
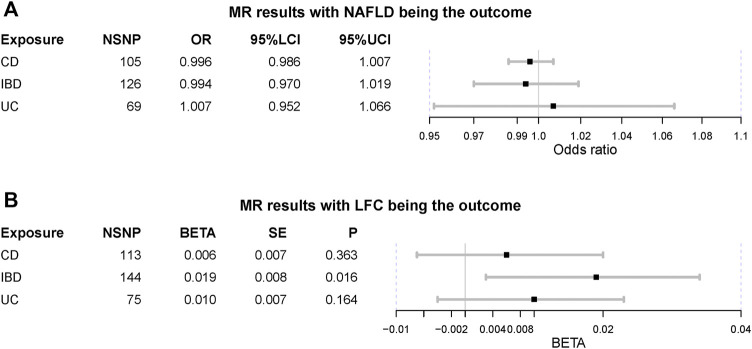
Mendelian randomization (MR) results where non-alcoholic fatty liver disease and liver fat content as the outcomes. NAFLD is non-alcoholic fatty liver disease; LFC is liver fat content; CD is Crohn’s disease; IBD is inflammatory bowel disease; UC is ulcerative colitis; NSNP is the number of single nucleotide polymorphisms used in MR analysis; OR is odds ratio; 95% LCI is the lower limit of 95% confidence interval of OR/BETA; 95% UCI is the upper limit of 95% confidence interval of OR/BETA; SE is standard error; P is the *p*-value of OR/BETA. BETA is for the continuous variable LFC.

Preliminarily, genetic predisposition to IBD could not elevate the risk of NAFLD (OR = 0.994 [0.970, 1.019], IVW *p*-value = 0.645), including two subtypes of IBD as UC (OR = 1.007 [0.952, 1.066], IVW *p*-value = 0.800) and CD (OR = 0.996 [0.986, 1.007], IVW *p*-value = 0.491) (Figure 2). After adjusting for BMI and T2D, the genetic predisposition to IBD could not affect the risk of NAFLD (OR = 1.057 [0.993, 1.125], IVW *p*-value = 0.082). Similar results were obtained for UC and CD in MVMR analysis as well (IVW *p*-value > 0.05). However, we observed a slight causal effect of IBD on LFC where genetically-elevated risk of IBD could lead to liver fat accumulation (beta = 0.019, se = 0.008, IVW *p*-value = 0.016). Considering LFC is a continuous variable, we used beta value to represent the effect size. It should be noted that the causal effect of IBD on LFC turned insignificant after Bonferroni correction (Bonferroni-corrected IVW *p*-value = 0.096). Besides, genetically-driven UC (beta = 0.010, se = 0.007, IVW *p*-value = 0.164) and CD (beta = 0.006, se = 0.007, IVW *p*-value = 0.363) could not alter LFC either (Figure 2). The impact of genetic predisposition to IBD on LFC was insignificant after adjusting for BMI and T2D (beta = -0.018, se = 0.010, *p*-value = 0.065). The MVMR results were similar in UC-LFC and CD-LFC associations (IVW *p*-value > 0.05). All pairs of causal association were insignificant in the MR-Egger regression and weighted-median method (Table 2). Although slight heterogeneity was detected for CD-NAFLD, CD-LFC and IBD-LFC pairs, the conclusions still held after removal of outliers. Also, genetic liability to CD could not affect the risk of NAFLD yet after correcting horizontal pleiotropy (OR = 0.024 [0.792, 1.008], MR-Egger *p*-value = 0.070).

**TABLE 2:** MR results of MR-Egger regression and weighted-median method.

			MR-Egger				Weighted-Median				p_heterogeneity_	P_pleiotropy_
Exposure	Outcome	NSNP	OR (BETA)	95%LCI	95%UCI	P	OR (BETA)	95%LCI	95%UCI	P		
UC	NAFLD	69	0.922	0.770	1.105	0.383	0.992	0.925	1.064	0.817	0.188	0.481
CD	NAFLD	105	0.024	0.792	1.008	0.070	0.020	0.990	1.006	0.655	0.016	0.038
IBD	NAFLD	126	0.947	0.822	1.091	0.455	1.004	0.978	1.030	0.785	0.063	0.499
UC	LFC	75	0.013	−0.023	0.049	0.487	0.012	−0.008	0.032	0.224	0.308	0.847
CD	LFC	113	0.032	−0.003	0.067	0.077	0.009	−0.009	0.026	0.327	0.015	0.253
IBD	LFC	144	0.024	−0.017	0.064	0.251	0.020	−0.002	0.042	0.080	0.000	0.797
NAFLD	UC	3	1.108	0.865	1.421	0.566	0.966	0.912	1.023	0.236	0.065	0.430
NAFLD	CD	3	1.039	0.872	1.238	0.741	0.987	0.936	1.041	0.635	0.483	0.635
NAFLD	IBD	3	1.067	0.934	1.220	0.513	0.979	0.938	1.021	0.321	0.318	0.373
LFC	UC	8	1.046*	0.883	1.239	0.622	0.976*	0.841	1.133	0.751	0.313	0.240
LFC	CD	11	1.118*	0.885	1.412	0.375	0.977*	0.861	1.109	0.718	0.002	0.076
LFC	IBD	10	1.055*	0.914	1.216	0.486	0.961*	0.875	1.057	0.415	0.103	0.110

UC, ulcerative colitis; CD, Crohn’s disease; IBD, inflammatory bowel disease; NAFLD, non-alcoholic fatty liver disease; LFC, liver fat content; NSNP, the number of single nucleotide polymorphism; OR, odds ratio; 95%LCI, lower limit of 95% confidence interval; 95%UCI, upper limit of 95% confidence interval; P, *p*-value of OR(BETA); P_heterogeneity_, *p*-value of heterogeneity test from Cochrane’s Q value; P_pleiotropy_, *p*-value of pleiotropy test from MR-Egger intercept.

Please note “*” represent the BETA, for the continuous variable LFC.

**FIGURE 3:**
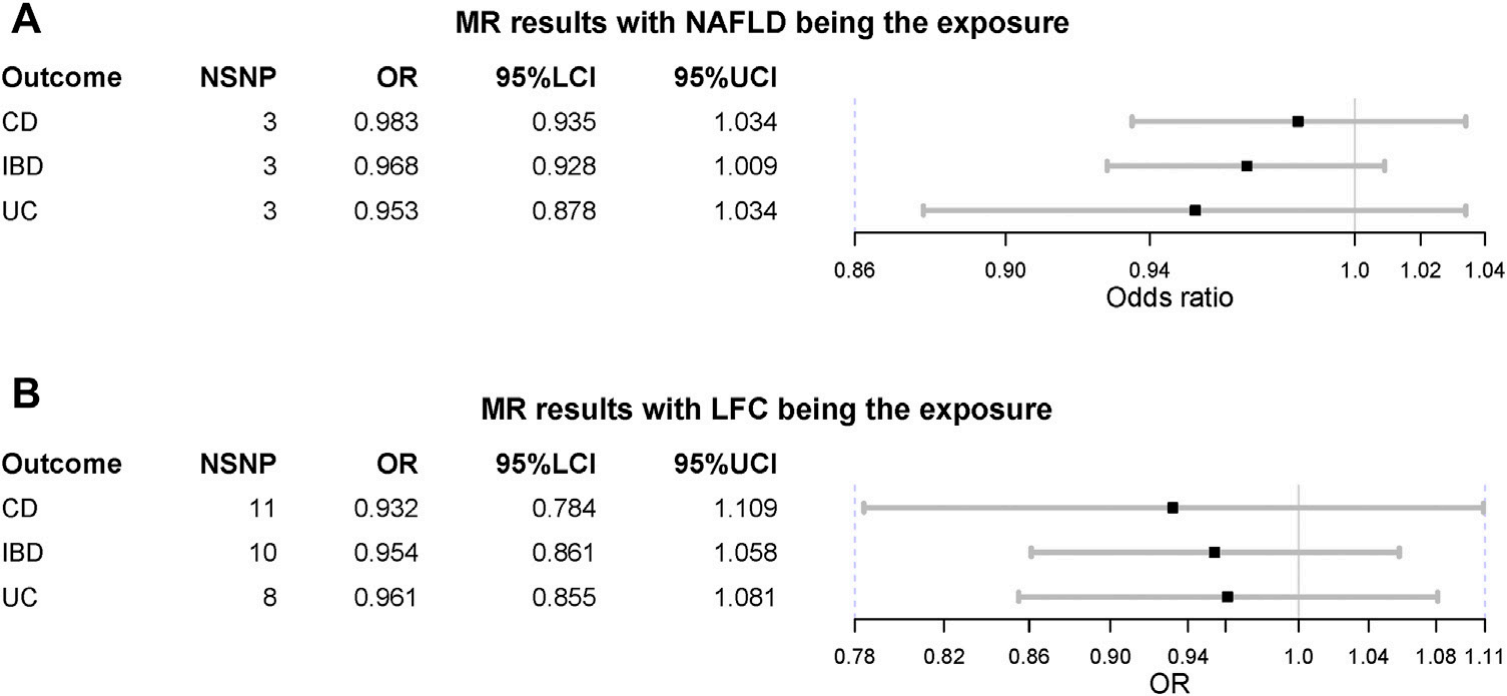
Mendelian randomization (MR) results where non-alcoholic fatty liver disease and liver fat content as the exposures. NAFLD is non-alcoholic fatty liver disease; LFC is liver fat content; CD is Crohn’s disease; IBD is inflammatory bowel disease; UC is ulcerative colitis; NSNP is the number of single nucleotide polymorphisms used in MR analysis; OR is odds ratio; 95% LCI is the lower limit of 95% confidence interval of OR; 95% UCI is the upper limit of 95% confidence interval of OR; P is the *p*-value of OR.

When treating NAFLD as the exposure, no causal association was detected, including IBD (OR = 0.968 [0.928, 1.009], IVW *p*-value = 0.123)**,** UC (OR = 0.953 [0.878, 1.034], IVW *p*-value = 0.247) and CD (OR = 0.983 [0.935,1.034], IVW *p*-value = 0.516) (Figure 3). The MVMR suggested there was no causation between NAFLD and IBD (UC and CD) after adjusting for BMI and T2D (IBD OR = 1.024 [0.983, 1.066], IVW *p*-value = 0.255). Also, the liver fat accumulation appeared not to affect the risk of IBD (OR = 0.954 [0.861, 1.058], IVW *p*-value = 0.373), UC (OR = 0.961 [0.855, 1.081], IVW *p*-value = 0.511) or CD (OR = 0.932 [0.784, 1.109], IVW *p*-value = 0.426). The MVMR suggested genetically-predicted LFC could not alter the risk of IBD, UC or CD after adjusting for BMI and T2D (IVW *p*-value > 0.05). No significant association was observed in MR-Egger regression and weighted-median method (Table 2). No horizontal pleiotropy was detected and there existed heterogeneity in NAFLD-UC and LFC-CD pairs. Also, the conclusions still held after removing outliers.

The MR CAUSE analysis indicated the causal model did not hold in estimating the causal associations abovementioned as all *p*-values of causal model were greater than 0.05 (Table 3). However, it should be paid attention to that the direction of gamma values from CASUE were all positive in estimating causal associations if treating NAFLD and LFC as the outcomes (gamma >0) while these gamma values turned negative if treating IBD, UC and CD as the outcomes (gamma <0). These results suggested the order of disease initiation might lead to opposite outcomes unexpectedly.

**TABLE 3:** Results of MR CAUSE analysis.

Exposure	Outcome	Model	Gamma	Eta	Q	P
UC	NAFLD	Sharing	0 (−0.01, 0.01)	−0.58 (−1.51, 0.43)	0.02 (0, 0.12)	0.937
		Causal		−0.54 (−1.64, 0.61)	0.02 (0, 0.15)	
CD	NAFLD	Sharing	0 (−0.01, 0.01)	−1.33 (−2, 0.02)	0.02 (0, 0.08)	0.842
		Causal		−1.3 (−2, 0.11)	0.02 (0, 0.09)	
IBD	NAFLD	Sharing	0 (−0.01, 0.01)	−1 (−1.55, −0.42)	0.06 (0.01, 0.12)	0.922
		Causal		−0.99 −1.57, −0.2)	0.06 (0.01, 0.13)	
UC	LFC	Sharing	0.01 (0, 0.02)	0.03 (-0.22, 0.33)	0.03 (0, 0.24)	0.523
		Causal		0 (−0.24, 0.31)	0.03 (0, 0.24)	
CD	LFC	Sharing	0.01 (0, 0.02)	0.02 (−0.45, 0.2)	0.03 (0, 0.23)	0.543
		Causal		−0.02 (−0.4, 0.18)	0.02 (0, 0.23)	
IBD	LFC	Sharing	0.01 (−0.01, 0.02)	0.03 (−0.31, 0.27)	0.03 (0, 0.23)	0.598
		Causal		−0.01 (−0.34, 0.23)	0.03 (0, 0.24)	
NAFLD	UC	Sharing	−0.03 (−0.06, 0)	−0.07 (−0.35, 0.32)	0.06 (0, 0.32)	0.168
		Causal		0.01 (−0.34, 0.42)	0.04 (0, 0.25)	
NAFLD	CD	Sharing	−0.02 (−0.06, 0.01)	−0.1 (−0.59, 0.34)	0.04 (0, 0.24)	0.543
		Causal		−0.05 (−0.57, 0.38)	0.04 (0, 0.24)	
NAFLD	IBD	Sharing	−0.03 (−0.05, 0)	−0.07 (−0.4, 0.3)	0.05 (0, 0.29)	0.127
		Causal		−0.01 (−0.42, 0.39)	0.03 (0, 0.24)	
LFC	UC	Sharing	−0.03 (−0.16, 0.09)	0.29 (−1.69, 3.81)	0.03 (0, 0.21)	0.880
		Causal		0.4 (−1.47, 3.74)	0.04 (0, 0.23)	
LFC	CD	Sharing	−0.02 (−0.14, 0.1)	−0.09 (−1.99, 2.14)	0.04 (0, 0.22)	0.985
		Causal		−0.04 (−1.88, 2.03)	0.04 (0, 0.23)	
LFC	IBD	Sharing	−0.04 (−0.12, 0.05)	−0.11 (−1.81, 2.77)	0.04 (0, 0.22)	0.823
		Causal		0 (−1.69, 2.75)	0.04 (0, 0.22)	

UC, ulcerative colitis; CD, Crohn’s disease; IBD, inflammatory bowel disease; NAFLD, non-alcoholic fatty liver disease; LFC, liver fat content. Model represents the type of two traits where “Sharing” means two traits have shared genetics and “Causal” means the exposure can causally affect the outcome. Gamma is the effect size of exposure on outcome; Eta is the effect size of correlated pleiotropy; Q represents the proportion of variants exhibiting correlated pleiotropy; P is the probability of accepting a sharing model.

After removal of outliers detected in MR-PRESSO, no SNP that might drive the results was identified in leave-one-out sensitivity analysis.

## Discussion

Although genetic liability to IBD might contribute to liver fat accumulation slightly, this MR study indicated there might be no causal link between NAFLD and IBD and it should be noted that the direction of NAFLD’s effect on IBD is negative, contrary to previous findings where NAFLD and IBD usually co-existed.

The observed causal effect of IBD on LFC might be a false positive one as this result was insignificant in both MR-Egger regression and weighted-median method. Besides, it was still insignificant in MR CAUSE analysis, a suitable method that can control false positive rate in MR analysis with consideration of both correlated and uncorrelated horizontal pleiotropy. However, we cannot completely rule out such causation as high-fat diet has impact on the quality of the intestinal barrier and the composition of the intestinal microbiome, influencing the pathogenesis of IBD (Ruemmele, 2016) and another study suggested LFC might be associated with UC as well (Jamali et al., 2017). Thus, further investigations should be carried out to elucidate such association.

As for the null associations, several reasons can be utilized to explain them. Metabolic syndrome, usually characterized by obesity, hyperglycemia, dyslipidemia and systemic hypertension, is currently the strongest risk factor of NAFLD (Friedman et al., 2018). Over 70% NAFLD patients are usually presented with high triglycerides (TG), high total cholesterol (TC), high low density lipoprotein cholesterol (LDL-C) and low high density lipoprotein cholesterol (HDL-C), most of which are primarily synthesized in liver, indicating dysregulated lipid metabolism in these patients, while the serum lipid profile is remarkably different in terms of serum TC and LDL-C in IBD patients at active stage, which can be rescued after intestinal surgery. Another study supported that less than 5% IBD patients are presented with dyslipidemia (Hoffmann et al., 2020), meanwhile, 25.0%–69.7% IBD patients at active stage developed malnutrition rather than obesity (Mijac et al., 2010). Therefore, we speculate that IBD brings out a specific metabolic state that might not be suitable for NAFLD.

Lean NAFLD is a special obesity resistant classification of NAFLD and believed to be with a distinct pathophysiological feature, characterized by higher serum secondary bile acid, increased expression of FGF19 and a shifted gut microbiota profile compared with non-lean NAFLD (Chen et al., 2020). IBD patients with NAFLD are often in absence of metabolic syndrome (Carr et al., 2017). There is still no report identifying the association between IBD and lean NAFLD. We cannot exclude that IBD and lean NAFLD have causal relationship, for no publicly available GWAS database can be utilized to address this problem until now.

Intriguingly, there was direct evidence illustrating that glucocorticoid for IBD could promote the initiation and progression of NAFLD instead of inhibiting NAFLD, and the use of azathioprine for CD was also determined as one risk factor for NAFLD (Woods et al., 2015). Similarly, bowel resection, a therapeutic strategy for severe IBD, was regarded as the risk factor for NAFLD in CD (Hoffmann et al., 2020), seemingly indicating inhibition of IBD could precipitate NAFLD. However, it is worthwhile to note that glucocorticoid as well as bowel surgery per se have the potential to result in liver steatosis or cholestasis, even without IBD history (Sasdelli et al., 2019). Thus, the observed co-existence of NAFLD and IBD might result from the effect of treatments, especially for the impact of IBD therapies on NAFLD (Restellini et al., 2017). Furthermore, Magri et al. reported there are no significant associations between NAFLD and IBD-related factors in IBD patients (Magrì et al., 2019). In a word, it is very difficult to demonstrate that IBD can contribute to the development of NAFLD based on previous studies.

On the other hand, accumulating evidence pointed to it that alteration of gut microbiota should have potential influence on the various risk factors of metabolic syndrome (Dabke et al., 2019). IBD is a chronic immunologically-mediated disease at the intersection of complex interactions between genetics, environment and gut microbiota (Ananthakrishnan, 2015), and gut microbiota have been reported to play an important role in pathogenesis of IBD (Franzosa et al., 2019). Metformin therapy for NAFLD could interact with gut microbiota (Vallianou et al., 2019) and so did the glucocorticoid therapy for IBD (Wu et al., 2018). Therefore, we postulate that gut microbiota may exert their effects on NAFLD and IBD concomitantly, and it can be comprehended as the pleiotropy of gut microbiota. As a result, NAFLD and IBD usually co-exist in clinical observation although there should be no causal relationship between them. Further investigations are needed to elucidate this hypothesis and corroborate our findings.

As no causal relationship was observed between NAFLD and IBD, it is still possible the underlying causal effect might be cancelled out due to opposite direct and indirect effects as omega 3 (*ω*3) fatty acids could alleviate intestinal inflammation (Marton et al., 2019). Therefore, it should be possible that the effects of “bad” lipids and “good” lipids can cancel out each other. Additionally, the negative results of MR study cannot completely rule out the causal relationship as the genetically-driven exposure cannot equals to the exposure and the negative results usually happen as the strict selection of IV.

As abovementioned, the pleiotropic effect of gut microbiota, impact of therapeutic treatments and opposite direct and indirect effects might help to explain the null causal relationship between NAFLD and IBD.

Our study has several strengths as follows: (Younossi et al., 2018) MR design was used to detect the causal relationship between and it could free this study from potential bias and reverse causation; (McHenry et al., 2019) both correlated and uncorrelated horizontal pleiotropy were controlled in this MR study; (McHenry et al., 2019) a bi-directional MR analysis was carried out to clarify the causation. However, some limitations should also be pointed out: (Younossi et al., 2018) horizontal pleiotropy should also be a major concern in MR study as various statistical methods fail to rule out horizontal pleiotropy caused by undetected biological mechanism; (Kaplan and Windsor, 2021) the proportion of NAFLD cases is relatively slow which might reduce the statistical power; (McHenry et al., 2019) the exclusion-restriction the selection might be violated as the binary phenotype was treated as the exposure due to data limitation; (Zou et al., 2019) the selection bias caused by competing risk factors could not be assessed as individual-level data was unavailable.

## Conclusion

This MR study ruled out the causal relationship between IBD and NAFLD, suggesting therapeutics targeting NAFLD might not work for IBD and vice versa.

## Data Availability

Publicly available datasets were analyzed in this study. This data can be found here: https://www.ebi.ac.uk/gwas/.
